# False-positive galactomannan assay in broncho-alveolar lavage after enteral nutrition solution inhalation: a case report

**DOI:** 10.1099/jmmcr.0.005116

**Published:** 2017-09-18

**Authors:** Olivier Lheureux, Isabel Montesinos, Olivier Taton, Martine Antoine, Jean-Charles Preiser, Joelle Nortier, Jacques Creteur, Frederique Jacobs, David Grimaldi

**Affiliations:** ^1^​ Department of Intensive Care, CUB - Erasme, Université Libre de Bruxelles (ULB), Route de Lennik 808, 1070 Brussels, Belgium; ^2^​ Department of Microbiology, CUB - Erasme, Université Libre de Bruxelles (ULB), Brussels, Belgium; ^3^​ Department of Pulmonary Medicine, CUB - Erasme, Université Libre de Bruxelles (ULB), Brussels, Belgium; ^4^​ Department of Cardiac Surgery, CUB - Erasme, Université Libre de Bruxelles (ULB), Brussels, Belgium; ^5^​ Department of Nephrology, CUB - Erasme, Université Libre de Bruxelles (ULB), Brussels, Belgium; ^6^​ Department of Infectious Diseases, CUB - Erasme, Université Libre de Bruxelles (ULB), Brussels, Belgium

**Keywords:** *Aspergillosis*, *Pneumonia*, feeding solution, nutritional support, immunosuppression, respiratory failure, fungal infection, voriconazole

## Abstract

**Introduction.** Diagnosis of invasive aspergillosis is challenging and the gold standard for definite diagnosis remains histopathological tissue examination. However, invasive procedures such as lung biopsy are often not feasible in critically ill patients. The detection of fungal cell wall components like *Aspergillus* galactomannan in broncho-alveolar lavage remains a key component of the diagnostic procedure. False-positive of the *Aspergillus* galactomannan assay is not frequent.

**Case presentation.** We report a case of positive galactomannan in broncho-alveolar lavage fluid after enteral nutrition aspiration without signs of invasive aspergillosis. Galactomannan was positive in the enteral nutrition solution.

**Conclusion.** Physicians should be aware of this previously unrecognized cause of false-positive galactomannan in broncho-alveolar fluid which can result in unnecessary treatments.

## Abbreviations

BAL, broncho-alveolar lavage; GM, galactomannan; IA, invasive aspergillosis; ICU, intensive care unit; VAP, ventilator-associated pneumonia.

## Introduction

We describe the first report of a false-positive broncho-alveolar galactomannan due to aspiration of enteral nutrition. As enteral nutrition solutions are prescribed daily in critically ill patients and aspiration pneumonia is a common complication in intensive care, physicians should consider this possibility in case of broncho-alveolar lavage positive galactomannan assay under artificial enteral feeding.

We believe this finding will be of great interest to microbiologists and physicians, especially to those in contact with patients in intensive care.

## Case report

A 65-year-old man was admitted in our intensive care unit (ICU) for pneumonia with septic shock. He was a heart transplant recipient since 1998 and had a renal graft in 2015 because of immunosuppressive treatment toxicity. Current immunosuppressive therapy consisted of methylprednisolone, mycophenolate mofetil and cyclosporine. Septic shock was associated with acute renal failure and severe acute respiratory distress syndrome requiring mechanical ventilation. No infectious pathogen was identified. The shock resolved in 4 days but respiratory conditions, only slowly improved, and the presence of a severe ICU-acquired weakness required a tracheotomy after 10 days of mechanical ventilation. The ICU stay was further complicated by a ventilator-associated pneumonia (VAP) due to *Pseudomonas aeruginosa*, a primary *Enterococcus faecalis* bacteremia and a severe herpetic stomatitis. A new respiratory deterioration at day 23 led to suspicion of a new episode of VAP treated by meropenem. Bronchoscopy revealed an important aspiration of enteral nutrition solution (Nutrison Protein Plus multifibre; Nutricia) due to incorrect position of the nasogastric tube. The procedure was followed by a pneumothorax, with pleural effusion, which required chest tube drainage. The pleural liquid had the same macroscopic aspect as the enteral nutrition solution. *Aspergillus* galactomannan (GM) assays performed in broncho-alveolar lavage (BAL) and pleural fluid, using the one-stage commercialized immunoenzymatic sandwich microplate assay (Platelia Aspergillus Ag; Bio-Rad), were highly positive in both fluids [optical density (OD) index 4.6 and >6, respectively; cut-off value >1]. Serum GM OD index was 0.08 (cut-off value >0.50) and fungal cultures of both BAL and pleural fluid remained sterile. We initiated voriconazole treatment and completed the work up with chest and sinus CT scans; both showed no signs suggestive of invasive fungal infection (see supplementary material). Considering these inconsistent findings for an invasive aspergillosis (IA), we performed *Aspergillus* GM detection in the enteral nutrition solution, which proved highly positive (index >6) whereas fungal culture remained negative. We therefore tested two other commercialized enteral nutrition solutions [Isosource standard fibre(Nestlé) and Fresubin original fibre (Fresenius Kabi)] also used in our institution and confirmed that all were also higher than the upper detection threshold (index >6). We therefore stopped voriconazole after 8 days of treatment. Evolution was favourable without any further fungal infection.

## Discussion

Species of the genus *Aspergillus* are ubiquitous in nature, and inhalation of infectious conidia is a common event that can give rise to various clinical conditions depending essentially on the host’s immunological status. IA occurs most frequently in the setting of severe immunosuppression (prolonged neutropenia, haematopoietic stem-cell transplantation, solid organ transplantation or acquired immune deficiency syndrome) [1].

Diagnosis of IA is challenging and the gold standard for definite diagnosis remains histopathological tissue examination. However, invasive procedures such as lung biopsy are often not feasible in patients with severe respiratory insufficiency and critical illness [2]. Despite the lack of sufficiently sensitive or specific non-invasive test to establish definite diagnosis, the detection of a fungal cell wall component like *Aspergillus* GM remains a key component of the diagnostic procedure. *Aspergillus* GM are polysaccharides released during growth of the fungus and are detected by ELISA in biological fluid. In the serum, a positive GM is a strong argument in favour of invasive aspergillosis but the test lacks sensitivity [3]. Therefore, detection of *Aspergillus* GM has been proposed in the BAL and seems to have a greater sensitivity. According to a meta-analysis, BAL GM sensitivity and specificity reach 92 and 96 %, respectively with a threshold of 1.5 µg l^−1^ in adult haematology patients [4]. Results are however lower in other immunosuppressed patient populations such as solid organ transplant recipients.

A false-positive result of the *Aspergillus* GM assay is not frequent. In BAL, false-positive GM assays have been observed in case of histoplasmosis [5] and in other mycoses [6, 7]. Indeed, some fungal species such as *Penicillium* and *Paecilomyces* have shown cross-reactivity with the rat EBA-2 monoclonal antibody used in our report, which has been characterized in previous studies [8]. False-positive BAL GM have been demonstrated after lavage with Plasmalyte (Baxter, Lessines, Belgium) and under treatment with piperacillin-tazobactam [9, 10]. Several reports incriminate GM-containing foods (pasta, rice, energy drinks, etc) as the source of positive serum GM through a chemotherapy-induced permeability of the intestinal mucosal barrier [11–13] but this was not reported in BAL fluids. A positive serum GM test linked to enteral feeding products has been mentioned exceptionally [14]. To the best of our knowledge, this is the first report of positive BAL GM assay induced by aspiration of enteral nutrition solution. Whether subclinical micro-aspiration of enteral nutrition solutions could also induce false-positive BAL GM should be further studied.

### Conclusion

This is the first report of a false-positive BAL GM due to aspiration of enteral nutrition. Physicians should consider this possibility in case of BAL positive GM assay under artificial enteral feeding.

## Supplementary Data

446 Supplementary File 1
