# Supplementary material for: False-positive galactomannan assay in broncho-alveolar lavage after enteral nutrition solution inhalation: a case report
Source: JMM Case Rep. 2017 Sep 18;4(9):e005116. doi: 10.1099/jmmcr.0.005116 (PMC5643004; doi:10.1099/jmmcr.0.005116)
Supplement: Supplementary File 1 [file jmmcr-4-5116-s001.pdf]

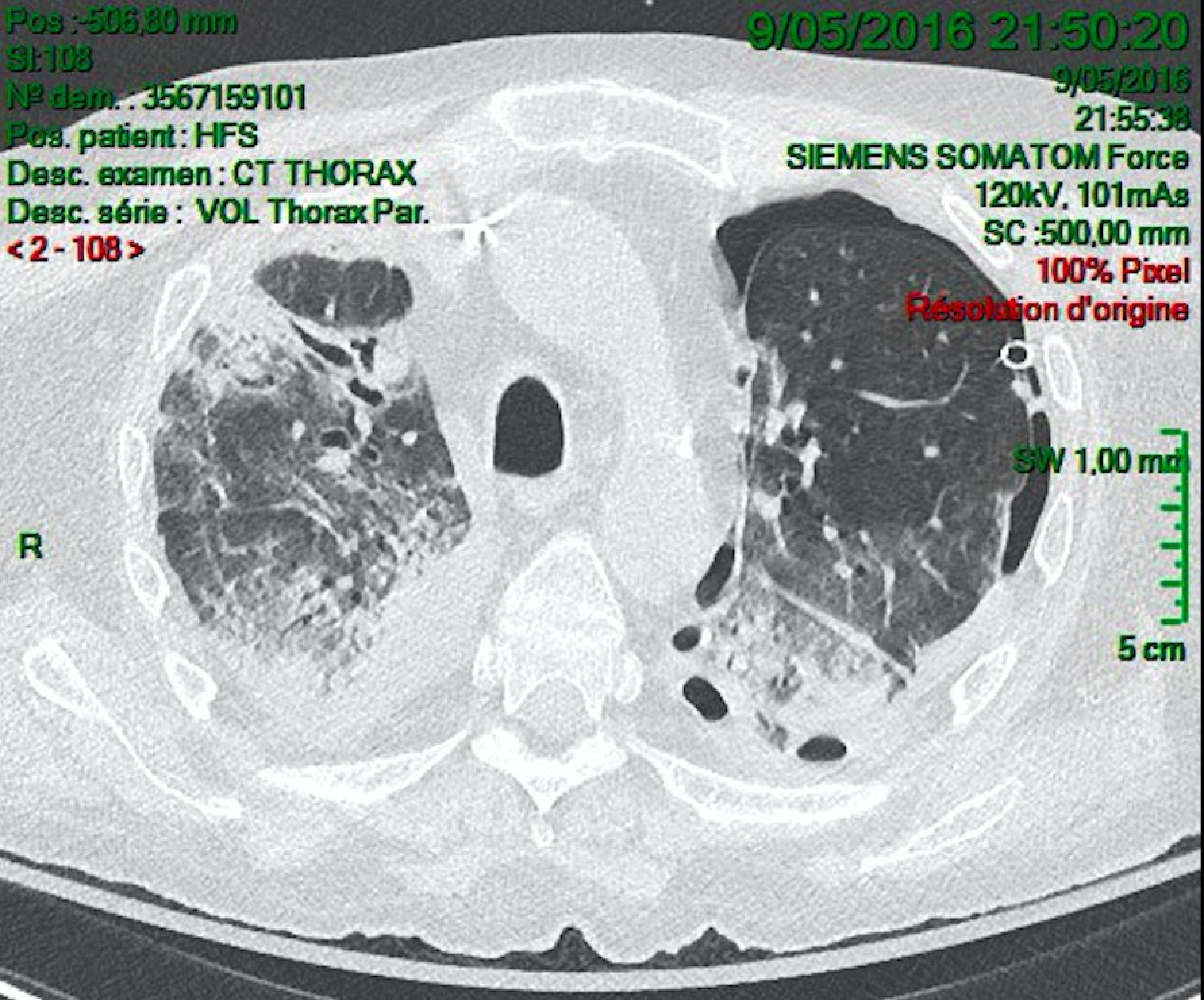
Pos :-506,80 mm
Sl:108
Nº dem. : 3567159101
Pos. patient : HFS
Desc. examen : CT THORAX
Desc. série : VOL Thorax Par.
< 2 - 108 >
9/05/2016 21:50:20
9/05/2016
21:55:38
SIEMENS SOMATOM Force
120kV, 101mAs
SC :500,00 mm
100% Pixel
Résolution d'origine
SW 1,00 mm
5 cm
R

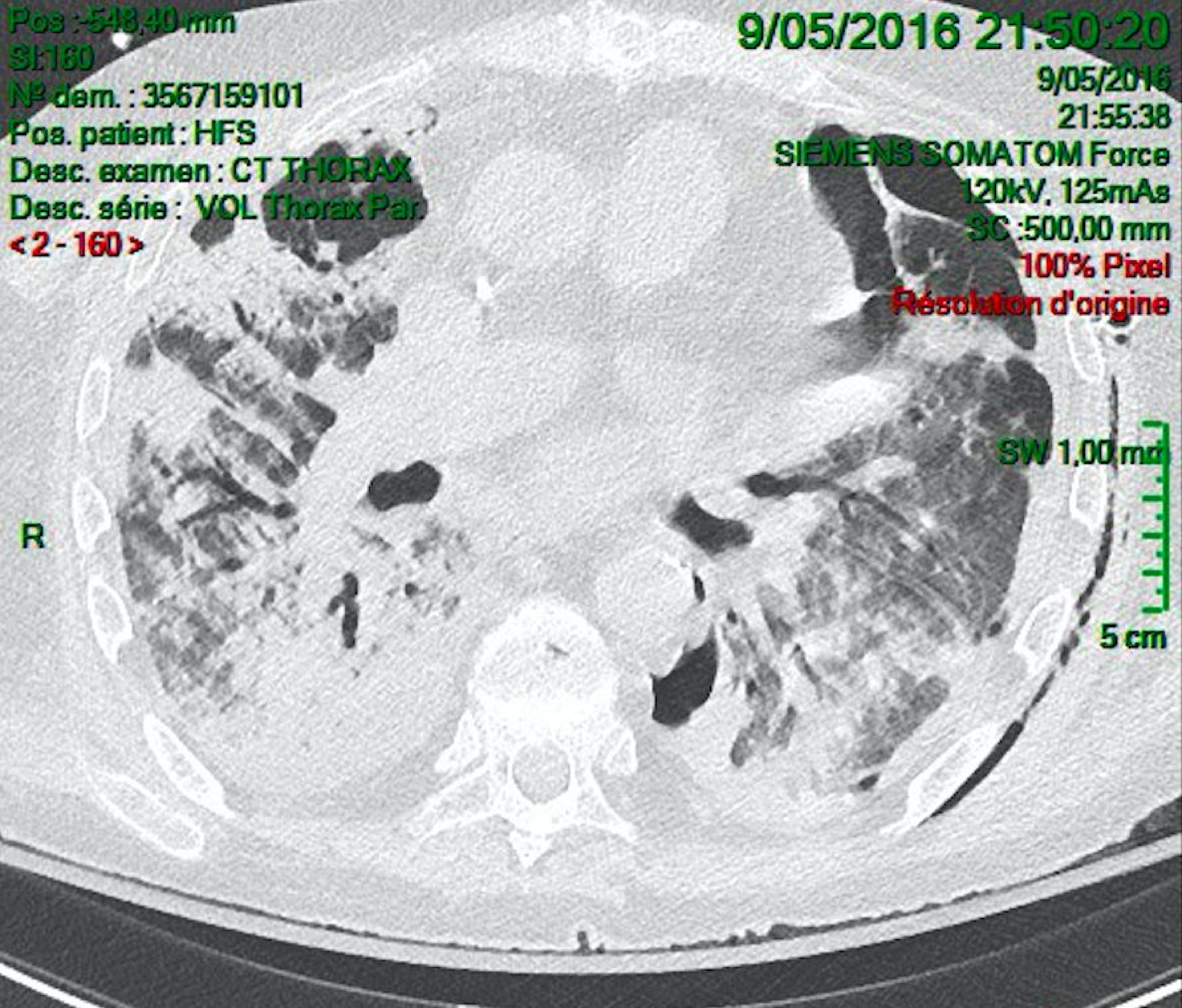

Pos :-548,40 mm
Sl:160
Nº dem. : 3567159101
Pos. patient : HFS
Desc. examen : CT THORAX
Desc. série : VOL Thorax Par.
< 2 - 160 >
9/05/2016 21:50:20
9/05/2016
21:55:38
SIEMENS SOMATOM Force
120kV, 125mAs
SC :500,00 mm
100% Pixel
Résolution d'origine
SW 1,00 m
R
5 cm

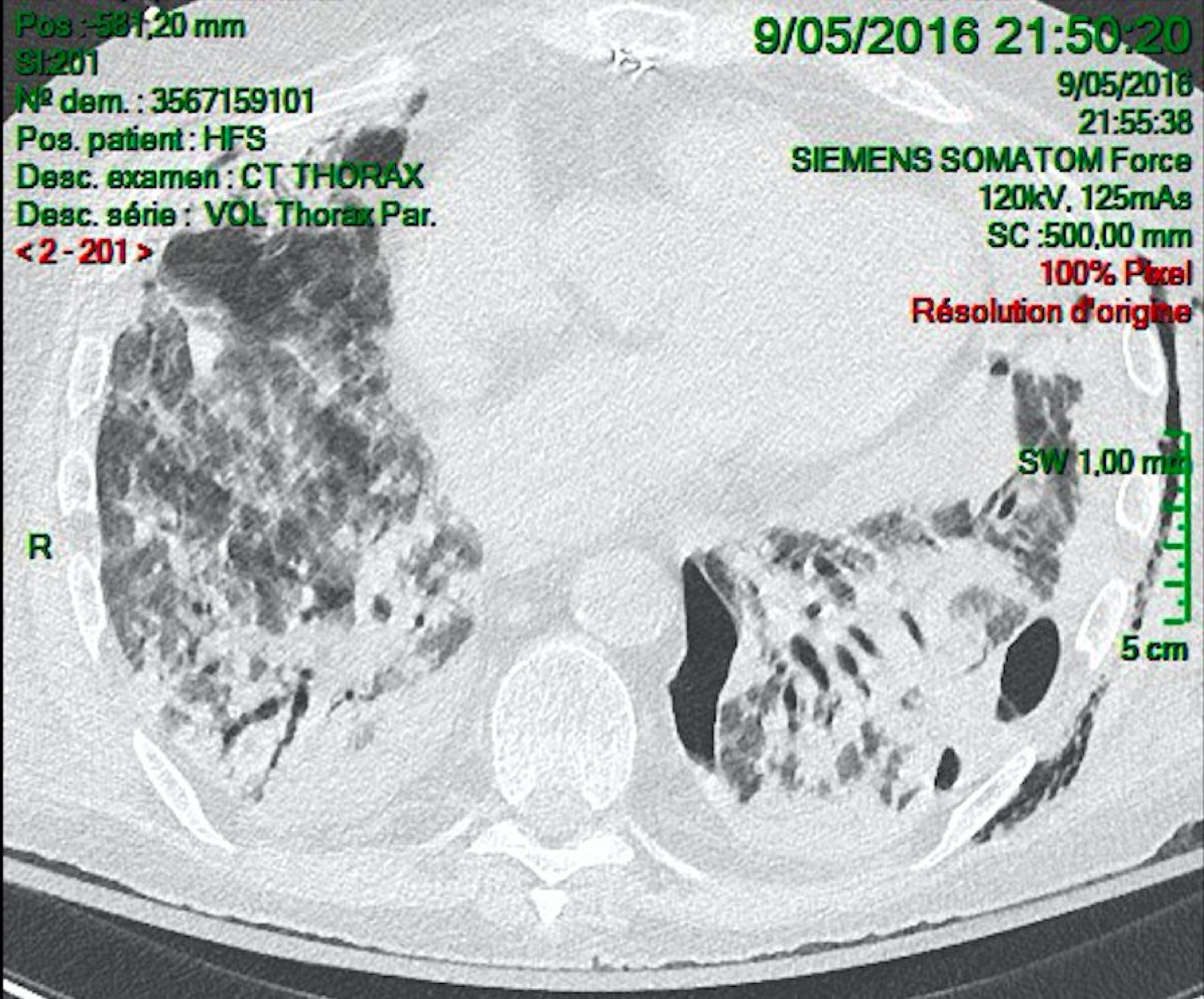
Pos :-581,20 mm
Sl:201
Nº dem. : 3567159101
Pos. patient : HFS
Desc. examen : CT THORAX
Desc. série : VOL Thorax Par.
< 2 - 201 >
9/05/2016 21:50:20
9/05/2016
21:55:38
SIEMENS SOMATOM Force
120kV, 125mAs
SC :500,00 mm
100% Pixel
Résolution d'origine
SW 1,00
R
5 cm
